# The soluble mannose receptor (sMR) is elevated in alcoholic liver disease and associated with disease severity, portal hypertension, and mortality in cirrhosis patients

**DOI:** 10.1371/journal.pone.0189345

**Published:** 2017-12-13

**Authors:** Thomas Damgaard Sandahl, Sidsel Hyldgaard Støy, Tea Lund Laursen, Sidsel Rødgaard-Hansen, Holger Jon Møller, Søren Møller, Hendrik Vilstrup, Henning Grønbæk

**Affiliations:** 1 Department of Hepatology & Gastroenterology, Aarhus University Hospital, Aarhus, Denmark; 2 Department of Clinical Biochemistry, Aarhus University Hospital, Aarhus, Denmark; 3 Department of Clinical Physiology and Nuclear Medicine, 239 Center for Functional and Diagnostic Imaging and Research, Faculty of Health Sciences Hvidovre Hospital, University of Copenhagen Copenhagen, Denmark; Medizinische Fakultat der RWTH Aachen, GERMANY

## Abstract

**Background and aims:**

Hepatic macrophages (Kupffer cells) are involved in the immunopathology of alcoholic liver disease (ALD). The mannose receptor (MR, CD206), expressed primarily by macrophages, mediates endocytosis, antigen presentation and T-cell activation. A soluble form, sMR, has recently been identified in humans.

*We aimed* to study plasma sMR levels and its correlation with disease severity and survival in ALD patients.

**Methods:**

We included 50 patients with alcoholic hepatitis (AH), 68 alcoholic cirrhosis (AC) patients (Child-Pugh A (23), B (24), C (21)), and 21 healthy controls (HC). Liver status was described by the Glasgow Alcoholic Hepatitis Score (GAHS), Child-Pugh (CP) and MELD-scores, and in AC patients the hepatic venous pressure gradient (HVPG) was measured by liver vein catheterisation. We used Kaplan-Meier statistics for short-term survival (84-days) in AH patients and long-term (4 years) in AC patients. We measured plasma sMR by ELISA.

**Results:**

Median sMR concentrations were significantly elevated in AH 1.32(IQR:0.69) and AC 0.46(0.5) compared to HC 0.2(0.06) mg/L; p<0.001 and increased in a stepwise manner with the CP-score (p<0.001). In AC sMR predicted portal hypertension (HVPG ≥10 mmHg) with an area under the Receiver Operator Characteristics curve of 0.86 and a high sMR cut-off (>0.43 mg/l) was associated with increased mortality (p = 0.005).

**Conclusion:**

The soluble mannose receptor is elevated in alcoholic liver disease, especially in patients with AH. Its blood level predicts portal hypertension and long-term mortality in AC patients.

## Introduction

The immunopathology of alcoholic liver disease (ALD) is incompletely understood and treatment options are limited. Therefore, identification of key pathophysiological components and possible targets for intervention is a priority in ALD.

Resident hepatic macrophages (Kupffer cells) are central in the current immunopathological understanding of ALD and we have previously shown that soluble CD163, the hemoglobin-haptoglobin scavenger receptor and a specific marker of macrophage activation, is highly elevated in ALD and originates from Kupffer cells[1–3]. This study focuses on the functionally different macrophage receptor, the mannose receptor (MR, CD206, MRC1) and its relation to clinical endpoints in ALD. The MR is expressed primarily by subsets of macrophages and by the comparatively scarcer dendritic cells, a soluble form (sMR) has recently been identified in humans [4]. MR is a pattern recognition receptor with a broad range of ligands including mannose, sulphated carbohydrates and collagen. The functions of the mannose receptor include endocytosis of microorganisms as well as facilitation of antigen presentation and subsequent induction of the associated immune responses[5, 6]. Furthermore, sMR has a metabolic role in the clearance of glycoproteins with degradation of endogenous glycoproteins such as β-glucuronidase and procollagen, which are up-regulated during inflammation[7]. In mice macrophages increase expression of the MR and its soluble form upon stimulation with IL-4 and IL-10, but the exact mechanism of sMR release in humans is unknown[8, 9]. sMR levels are elevated in critically ill patients receiving intensive care and predict survival in patients with pneumococcal bacteraemia and patients with acute on chronic liver failure[10–12].

In ALD, Kupffer cell activation likely occurs as a consequence of bacterial translocation from the gut to the liver. Early animal studies have shown that ethanol-mediated leakage of gut endotoxins to the liver leads to activation of Kupffer cells and initiation of hepatic inflammation [13–15]. This mechanism seems to be important in humans as well, and both increased gut permeability as well as increased serum levels of bacteria and bacterial products have been demonstrated in human ALD [1, 16–20]. The exact role of Kupffer cells remains controversial with different studies showing booth beneficial and deleterious effects of the cells. [21, 22]

This study was conducted to pursue our previous findings of Kupffer cell activation in ALD, and to obtain corroborative and functionally independent confirmation of it´s role. We hypothesize that plasma concentrations of sMR are higher in ALD patients compared with healthy controls, that sMR is associated with ALD disease severity and portal hypertension, and that it can predict clinical endpoints. For this purpose we investigated plasma levels of sMR in ALD patients with alcoholic hepatitis, alcoholic cirrhosis, and healthy controls and measured the hepatic venous pressure gradient (HVPG) by liver vein catheterization, and investigated the association with short- and long-term survival in AH and AC patients,

## Methods

### Patients

We conducted a cohort study on three groups: 1) 50 patients admitted with acute AH; 2) 68 patients with alcoholic cirrhosis (AC) with different CP-score; and 3) 21 healthy persons with no history of liver or other diseases.

The AH patients were consecutively included using the following inclusion criteria: A first time diagnosis of AH by a combination of physical and laboratory criteria: A history of excessive alcohol ingestion (10 units or more per day) until at least three weeks before admission; acute jaundice (developed over at most 2 weeks, serum bilirubin > 80 μmol/L). The diagnosis was verified by liver biopsy in 10 patients. Exclusion criteria were: viral hepatitis, autoimmune liver disease, bile duct obstruction, liver tumors or any other cancer, presence of an infectious focus (either clinically assessed or based on chest x-ray, urine samples or ascites puncture), age below 18 or above 75 years, ongoing gastrointestinal bleeding or bleeding within the previous three months, or any prior immune-modulating therapy. Patients were recruited from four hospitals in the Aarhus region in Denmark. The patients were stratified according to the Glasgow Alcoholic Hepatitis Score (GAHS) [23]. Those with a GAHS < 9 were given moderate hyper-alimentation (a daily energy consumption of 35–49 Kcal/kg body weight and a daily protein intake of 1.2–1.5 g/kg) and standard supportive medical care, and those with a GAHS ≥ 9 were treated orally with pentoxifylline 400 mg TID (Trental®, Sanofi Paris, France) according to the national guidelines at the time of the study. Clinical status was also assessed according to the Model for End-stage Liver Disease (MELD) and the Child-Pugh score[24–26]. The infectious status at baseline was recorded. The patients were followed-up for 30 days and blood samples were collected at study entry and at day 7, 14, 21, & 30. Entry blood samples were secured before initiation of treatment. The mortality by day 84 (12-weeks) was recorded in the AH patients. Baseline data and baseline sMR levels from this cohort have previously been reported [1, 4]. No patients received blood transfusions during the study period.

The AC patients (n = 68 Child-Pugh A: n = 23, B: n = 24, C: n = 21) were all set up for clinical routine liver vein catheterization. The HVPG was determined using Swan-Ganz balloon catheters as previously described [27]. Blood was collected during catheterisation. Baseline data has partly been reported previously [2]. None of the patients had signs of alcoholic hepatitis, infection or had fever at examination and none of the patients were treated with antibiotics. The diagnosis of cirrhosis was based on a combination of clinical and biochemical parameters as well as ultrasound or CT-scan. The patients underwent routine clinical assessment of the severity of liver disease by CP-class and MELD score. The galactose elimination capacity (GEC) as a measure of metabolic liver function was determined as previously described [28]. Clinically significant portal hypertension (CSPH) was defined by a HVPG ≥10 mmHg measured during hepatic vein catheterization.

Survival data was collected after four years.

The 21 control persons were referred for hemodynamic investigation to exclude splanchnic perfusion disorders, for which no evidence was found. The HVPG was determined by the same procedure as in the AC patients.

All participants gave informed consent in accordance with the Helsinki Declaration, and the study was approved by the National Committee on Health Research Ethics and the Danish Data Protection Agency (J-No. 2008-41-2020) & (J.nr. 2008-41-2836).

### Biochemical analysis of sMR

The plasma concentration of sMR was determined by ELISA as previously described.[4]

The maximum inter-assay coefficient of variation was 15%, and the intra-assay coefficient of variation was below 10%.

Biochemical analysis of soluble cytokines interleukin 17a (IL-17a) & interleukin 22 (IL-22)

IL-22 has a protective role in AH, and to investigate the relationship between sMR and other parts of the immune system we measured IL-22 and the related pro-inflammatory cytokine IL-17a in 20 randomly selected AH patients.

The plasma concentrations of IL-17A, IL-22 were measured with ELISA kits (eBioscience) as previously described[29]. The samples and standards were analysed in duplicate, and the average optical density, minus the average value of the blanks, was used to calculate the concentration of a given sample based on the standard curve. The lower detection limit was determined as the value of the average of the blanks plus 2 standard deviations, which resulted in cut-off values of 2.72 pg/ml (IL-17A), 9.04 pg/ml (IL-22). The values below the detection limit were assigned the cut-off value.

### Flow cytometry

To investigate whether peripheral monocytes express MR and therefore could be a source of sMR, we studied the MR expression using flow cytometry. 20 patients with AH, 10 patients with AC, and 10 HCs was studied. Peripheral blood mononuclear cells were isolated by Ficoll-Hypaque (GE Healthcare Bio-sciences, Uppsala, Sweden) from EDTA whole blood samples. Samples were stored at -140°C until en bloc analyses. Cells were thawed in PBS containing 20% heat-inactivated pooled human AB-serum and blocked with 10 μl heat-inactivated mouse serum (Invitrogen, Carlsbad, California, USA) before staining with optimized amounts of fluorescent-conjugated antibodies; CD14-APC (clone MFP9; BD Bioscience, San Diego, CA), CD206-FITC (clone 19.2; BD Bioscience, San Diego, CA). Relevant isotype controls were included. The samples were analysed within 24 hours of preparation using a FACSCanto instrument (BD Bioscience). Data analyses were carried out using FlowJo v10.1 (Tree Star Inc., Ashland, OR). We report the relative fluorescent intensity (MFI) of sMR on the CD14+ monocytes.

### Statistical analyses

We used the Kruskal-Wallis test followed by Mann-Whitney test to study differences between groups. The relationships between sMR and clinical and biochemical variables were analysed by Spearman's rank correlation. We examined if sMR was independently associated with CSPH by multiple logistic regression analyses, including the MELD-score, GEC and C-reactive protein (CRP) in the model.

We used Receiver Operator Characteristics (ROC) analyses to evaluate the overall capability of the sMR to predict clinically significant portal hypertension (CSPH) defined as a HVPG ≥10 mmHg. Using the ROC curve, a cut-off point was selected for predicting CSPH. The diagnostic accuracy of the cut-off was determined by calculating sensitivity, specificity, positive predictive value (PPV) and negative predictive value (NPV). Healthy persons were excluded for ROC-analysis. In the AH patients, an analysis of variance for repeated measurements with Greenhouse–Geisser correction for sphericity was performed for a comparison of protein levels over time. To ensure normally distributed variables, logarithmic transformation was used where appropriate. Histograms and Q–Q plots were used to check normality.

All data are expressed as median with inter-quartile range, and P-values ≤0.05 were considered statistically significant. STATA version 11.2 **®**StataCorp LP was used for the data analyses.

## Results

### Patients

Age and gender distributions were similar in the three study groups (Table 1). As expected, the patients with AH displayed a more severe liver dysfunction than the AC patients. No patient displayed signs of infection at study entry; however, eight AH patients (16%) developed blood culture positive infection during the 30-day follow up.

**Table 1:** Study entry characteristics of patients with alcoholic hepatitis and patients with stable alcoholic liver cirrhosis and healthy persons (Data are median (interquartile range)).

	Alcoholic Hepatitis		Alcoholic cirrhosis		Healthy Controls
Gender: F/M	16 /	34	16 /	52	7 / 13
Age (years)	53	(11) ++	58	(12) *	51 (8)
Weight (kg)	75.9	(25)	75	(25)	58 (25)
Height (cm)	174	(11) *	173	(12)	166 (16)
BMI	24	(6)	25	(5)	25 (8)
ALT (U/l)	47	(39)	39	(27)	30 (10)
Sodium (mmol/l)	132	(7) * ++	139	(6) *	143 (3)
Bilirubin (μmol/l)	283	(255) * ++	18	(14) *	7 (12)
Alkaline phosphatase (U/l)	203	(149) * ++	169	(127)	124 (116)
Hemoglobin (mmol/l)	6.7	(1)	7.4	(2)	NA
Creatinine (μmol/l)	64	(38) ++	78	(33)	72 (14)
INR	1.9	(0.7) * ++	1.3	(0.3) *	1.05 (0.1)
Albumin (g/l)	27	(8) * ++	34	(10) *	41 (7.7)
MELD	21	(11)	9	(6)	
GAHS	9	(2)			
Child Pugh Score	11	(2)	7	(4)	
Ascites (yes/no)	(31/19)		(37/31)		NO

ALT = Alanine aminotransferase, MELD = Model for End-stage Liver Disease, GAHS = Glasgow Alcoholic Hepatitis Score

INR = International Normalised Ratio.

*p<0.05 pt. vs controls

++p<0.05 AH. vs AC

### sMR was elevated in alcoholic liver disease

**Fig 1:**
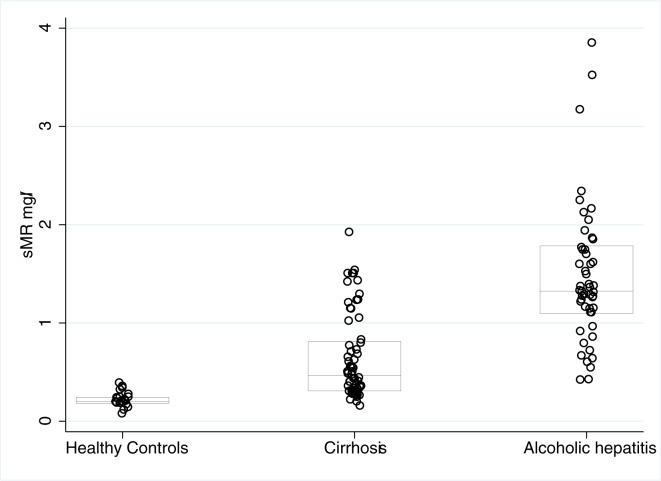
sMR levels in Alcoholic hepatitis, Cirrhosis & healthy control subjects.

**Fig 2:**
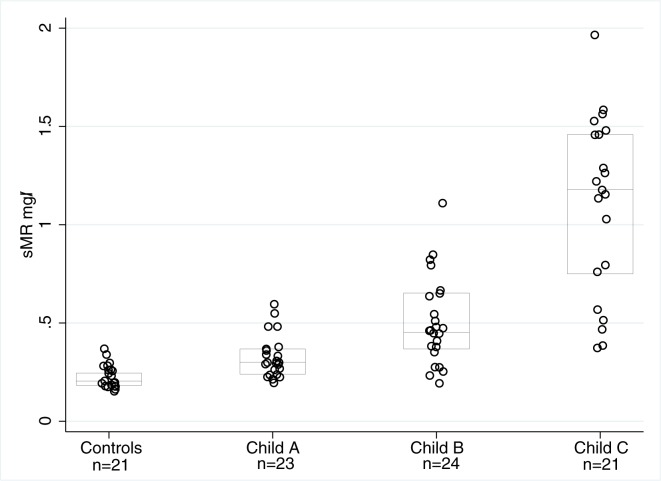
sMR in cirrhosis. sMR (mg/L) levels in healthy control subjects and patients with different degrees of liver cirrhosis. P < 0.05 controls compared to patients with liver cirrhosis. P < 0.05 CP-C compared to CP-A and CP-B.

At baseline, the plasma sMR concentration in the AH patients was approximately 3-fold higher than in the cirrhosis patients and nearly 7-fold higher than in the healthy persons (AH 1.32(0.69), AC 0.46(0.5), HC 0.2(0.06) mg/L; p < 0.001, Fig 1). In AC patients, the sMR concentrations increased in a stepwise manner according to the Child-Pugh score. (Fig 2, p<0.05) sMR levels were higher in patients with ascites compared to patients without ascites, and this was the case for both AH patients (1.5 vs 1.1 mg/L; p = 0.003) and AC patients (0.7 vs 0.3 mg/L; p<0.001).

**Fig 3:**
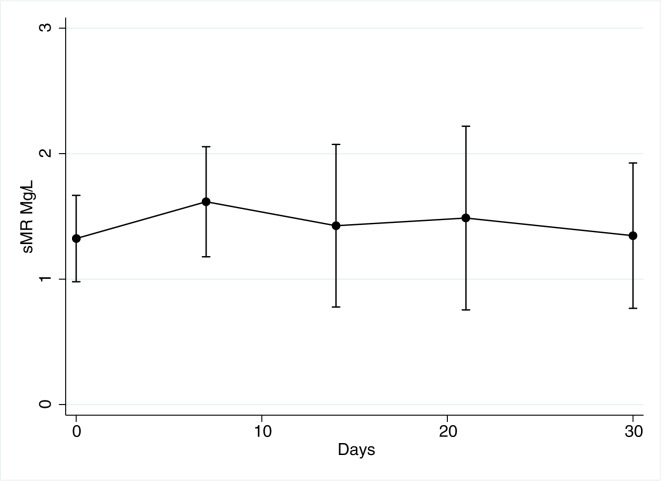
sMR in AH patients over time. sMR levels in Alcoholic hepatitis patients during the 30-day follow-up. (p = 0.07, repeated measures ANOVA).

**Fig 4:**
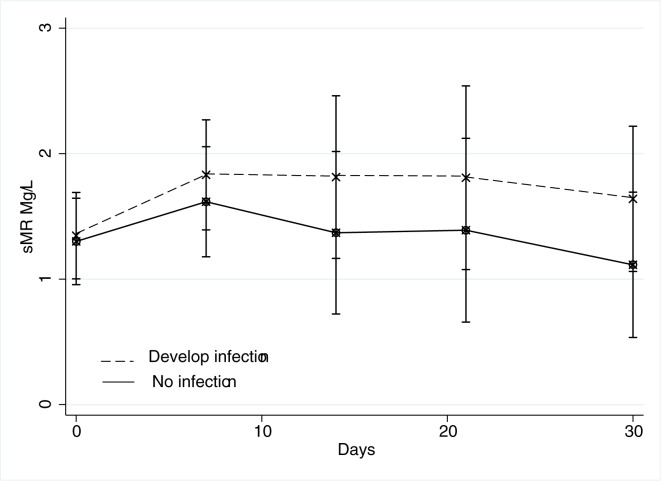
sMR in AH patients by infection. sMR levels in Alcoholic hepatitis patients during the 30-day follow-up allocated by patients who later developed infection n = 8 and patients who did not n = 42. (p = 0.07, repeated measures ANOVA).

In the AH patients, the sMR concentrations was unchanged during the 30-days follow-up period (p = 0.07; Fig 3). There was no difference at baseline in sMR levels between patients who later developed infection (and subsequently received antibiotic treatment) and those who did not (1.35 vs 1.30 mg/l; p = 0.99). However, sMR levels rose in patients who developed infection, but this rise in sMR level did not reach statistical significance (p = 0.33 repeated measures ANOVA, Fig 4). We observed no differences in sMR levels whether the patients were treated with Pentoxifylline or not (p = 0.9).

### Internal correlations

In AH patients, baseline analysis showed statistically significant correlations between sMR and Child-Pugh score (r = 0.41; p = 0.003), and albumin (r = 0.37; p = 0.009), but sMR did not correlate to any other clinical or biochemical measures including the GAHS score and cytokines IL-17a (mean 4.0 pg/ml (IQR 2.5) rho -0.2 p = 0.4) and IL-22 (mean 50 pg/ml (IQR 45) rho -0.13 p = 0.58)

At variance, in the AC patients we observed significant correlations between sMR and both clinical and biochemical parameters: The Child-Pugh score (r = 0.71; p < 0.001), the MELD score (r = 0.45; p = 0.002), albumin (r = 0.72; p < 0.001), INR (r = 0.46; p < 0.001), CRP (r = 0.52; p < 0.001), and bilirubin (r = 0.36; p = 0.003).

### Relationship between sMR and HVPG (alcoholic cirrhosis patients only)

sMR correlated positively with HVPG (r = 0.52, P<0.001) and sMR was independently associated with CSPH after adjustment for CRP, MELD-score and GEC, (p = 0.03) in multiple logistic regression analysis, Table 2.

**Table 2:** Multiple logistic regression analyses of the relationships between Clinically significant portal hypertension & sMR, CRP, GEC, and MELD-score.

			N = 60	
Dependent Variable = CSPH			Chi square = 0.0001	
Predictor variables	OR	Std. Error	p-Value	Coeficients
sMR	7379.9	29678.0	0.027	8.90
CRP	0.9	0.1	0.286	-0.06
MELD-score	1.3	0.2	0.076	0.23
GEC	0.5	0.4	0.413	-0.78

CSPH = Clinically significant portal hypertension; GEC = Galactose elimination capacity; CRP = C-Reactive Protein.

**Fig 5:**
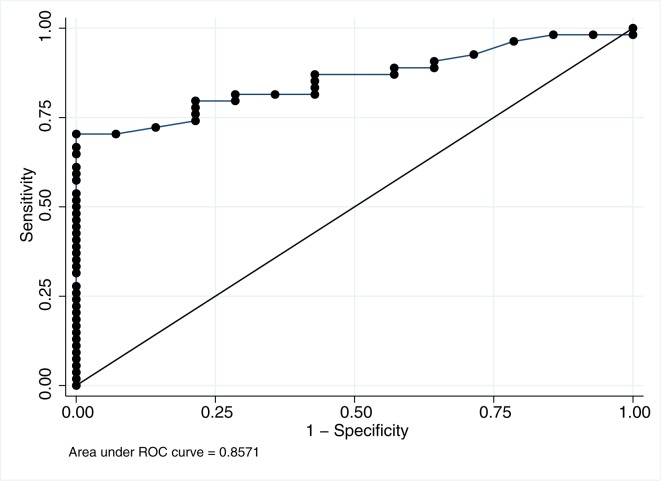
sMR as a predictor of portal hypertension. Receiver operating characteristic (ROC) analysis of sMR as a predictor for CSPH in patients with alcoholic cirrhosis.

The ROC analysis showed sMR to be a strong predictor of portal hypertension (AUC ROC = 0.86) (Fig 5). For further analysis we chose a cut-off for sMR at 0.43 mg/L equal to the upper normal level. At this cut-off sMR had a sensitivity of 70% and a specificity of 100% for the prediction of CSPH, corresponding to a positive predictive value of 100% and a negative predicted value of 46%.

### Mortality

The AH patients were followed up for 84-day mortality and within that period 11 of the 50 (22%) patients died. All but one of the diseased had a GAHS ≥ 9 on admission. We divided the patients into two groups using the median level of sMR (1.3 mg/L) and this analysis showed no difference in mortality between the groups (p = 0.46, log-rank test; data not shown).

**Fig 6:**
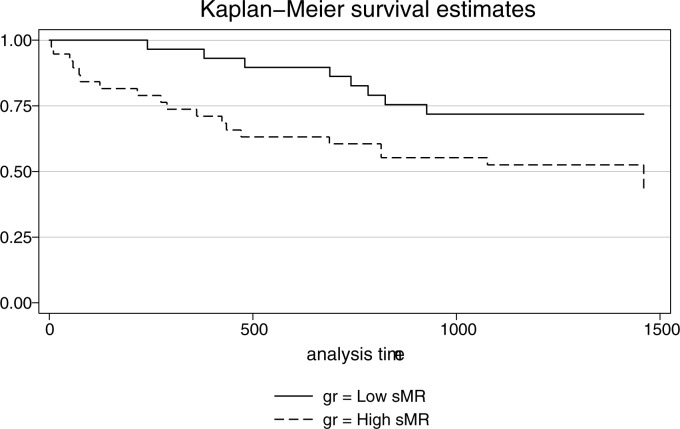
sMR and survival. Kaplan-Meier 4-year survival estimates in alcoholic cirrhosis patients according to the plasma level of sMR. The cut-off used was 0.43 (mg/L) (P = 0.03, Log-rank).

The alcoholic cirrhosis patients were followed up after four years and 41% (28/68) of the patients died. We divided the patients into two groups using the same sMR cut-off of 0.43 mg/Land the AC patients with a sMR above the cut-off had the higher mortality, 53% vs. 27% (p = 0.03, Fig 6).

### Blood monocyte MR & CD14 expression

There was an increase in the numbers of peripheral monocytes in patients with AH compared to both the healthy controls and the AC patients (p = 0.006, data not shown), but we detected no expression of membrane bound MR on CD14^+^ monocytes in neither patients or controls by flow cytometry.

## Discussion

The central finding of our study was the elevated plasma sMR in patients with alcoholic liver disease, most markedly in the patients with alcoholic hepatitis. In the cirrhosis patients, sMR rose with higher Child-Pugh score and with increasing portal pressure. Thus, the levels of sMR accurately predicted if they had clinically significant portal hypertension and their long-term mortality. These findings confirm and support our earlier reports based on sCD163 on the role of the macrophages in ALD, using a new and functionally alternative immune-pathological approach[30] [31].

The major strength of our study is the relevant number of well-characterized patients with ALD. We established the diagnosis of AH in our patients by using a combination of standard clinical and biochemical criteria in accordance with several clinical trials [23, 32]; and the diagnosis was confirmed by liver biopsy in a subset of patients. Likewise, the diagnosis of cirrhosis was made clinically by a combination of clinical, biochemical parameters as well as ultrasound or CT-scan. Interestingly, we could not detect and impact of pentoxifyllin treatment on sMR levels in the AH patients, and this possibly corresponds to the limited clinical impact of pentoxifyllin. It would be interesting to investigate the impact of steroid treatment on sMR levels in AH patients.

A soluble form of the human MR was demonstrated in *in vitro* experiments in 1999, but it was not till recently that sMR was detected in human blood samples [4, 33]. As a consequence, few studies have investigated sMR in human disease. These studies demonstrated elevated sMR in both critically ill sepsis patients and hepatitis C cirrhosis patients with levels in hepatitis C cirrhosis patients comparable to our AC patients [12, 34]). Of interest, patients with sepsis and liver disease had the highest sMR levels. Recently it was demonstrated that sMR is elevated in patients with acute-on-chronic liver disease (ACLF), and in relation to disease severity; and further, that high sMR levels was an independent predictor for long-term survival in cirrhosis patients without ACLF. Indeed, the addition of sMR to the CLIF-C Acute Decompensation score significantly improved the prognostic performance of the original score[10, 35].

Recent alcohol intake may moderately increase sMR, and it is a limitation that we do not have such information prior to study entry. However, if alcohol should be important we would expect a rapid decline in sMR during the 30-day follow-up of the AH patients. More likely, the florid hepatic inflammation in AH has passed a threshold where alcohol cessation alone will not be enough to dampen inflammation. It is also a limitation that we do not know to which extent the dendritic cells contribute to the generation of sMR, and what pathophysiological implications this might have. However, in comparison to Kupffer cells, dendritic cells are sparse in the liver, and primarily located to the portal regions [36]. Very little is known about clearance of sMR but renal excretion is unlikely owing to the molecular size of the protein (approximately 170 KD). Decreased hepatic metabolism because of shunting might contribute to the elevated levels seen in this study, but we don’t have any specific data to support this. We believe that the origin of the high levels of sMR measured in our study is the liver and predominantly the Kupffer cells. In line with a previous study we found that blood monocytes did not express MR and are an unlikely source of sMR [12].

The functions of sMR are not completely understood, but the soluble part of the receptor retains its binding capacity and thus may serve as a shuttle targeting circulating antigens to the lymphoid organs *[37, 38]*. There seems to be a slight constitutive shedding of the MR and certain inflammatory mediators elicit extensive shedding. However, LPS is not among these mediators and conversely appears to decrease the expression and shedding of the MR[33]. This is noteworthy because translocated bacteria and LPS presented to the liver is believed to play a key role in the pathogenesis of ALD. Our results with an increased sMR thus cast doubts on the importance of this mechanism. On the other hand sCD163, another marker of macrophage activation, increases its expression and shedding by LPS stimulation and is markedly increased in ALD[1, 39]. These seemingly contradictory results may be reconciled by the assumption that hepatic macrophage activation is effectuated by a variety of stimuli among which LPS is an identified part.

The diversity of the stimulatory pathways notwithstanding, our study supports the occurrence of Kupffer cell activation in ALD and confirm that this may play a role in the immunopathology which may also be corroborated by the repeatedly demonstrated associations of macrophage activation markers, in this case sMR, with clinically important endpoints. Thus, in the AC patients, the ROC-analysis showed a very high positive predictive value of an increased sMR for clinically significant portal hypertension (>10 mmHg). It is well known that hepatic inflammation leads to portal hypertension and we believe the sMR correlation with CSPH mirrors this. Several non-invasive methods for identifying CSPH are under investigation and sMR measurements may contribute to this field alone or in combinations[30]. Given its properties as an endocytic receptor, MR might also be a potential target for intervention with antibody bound compounds. Such proof of concept already exists for the endocytic macrophage receptor CD163. [40, 41]

In conclusion, the blood concentration of the soluble mannose receptor sMR is elevated in ALD, especially in patients with AH. Its level is independently associated with severe portal hypertension and long-term mortality in cirrhosis. Our study confirms and expands our previous findings of macrophage activation in ALD by a different biological approach and possibly motivates intervention studies.

## Supporting information

S1 File Smr data.dta. Project data stata format.
(DTA)

## Abréviations

sMR: Soluble (s) Mannose Receptor
ALD: Alcoholic liver disease
AH: Alcoholic hepatitis
AC: Alcoholic cirrhosis
HC: Healthy controls
GAHS: Glasgow Alcoholic Hepatitis Score
CP: Child-Pugh
MELD: Model for end stage liver disease
HVPG: Hepatic venous pressure gradient
ELISA: Enzyme-linked immunosorbent assay
GEC: Galactose elimination capacity
CSPH: Clinically significant portal hypertension
EDTA: Ethylenediaminetetraacetic acid
PBS: Phosphate-buffered saline
MFI: Relative fluorescent intensity
CRP: C-reactive protein
ROC: Receiver Operator Characteristics
PPV: Positive predictive value
NPV: Negative predictive value
ACLF: Acute-on-chronic liver disease
LPS: Lipopolysaccharides
